# Immune Signaling Kinases in Amyotrophic Lateral Sclerosis (ALS) and Frontotemporal Dementia (FTD)

**DOI:** 10.3390/ijms222413280

**Published:** 2021-12-10

**Authors:** Raquel García-García, Laura Martín-Herrero, Laura Blanca-Pariente, Jesús Pérez-Cabello, Cintia Roodveldt

**Affiliations:** 1 CABIMER-Andalusian Center for Molecular Biology & Regenerative Medicine, University Seville-CSIC-UPO, 41092 Seville, Spain; raquel.garcia@cabimer.es (R.G.-G.); laumarher2@alum.us.es (L.M.-H.); lauramariabp@hotmail.es (L.B.-P.); jesus.perez@cabimer.es (J.P.-C.); 2 Department of Medical Biochemistry, Molecular Biology and Immunology, University of Seville, 41009 Seville, Spain

**Keywords:** amyotrophic lateral sclerosis (ALS), frontotemporal dementia (FTD), kinase, neuroinflammation, immune, signaling, neurodegeneration, inhibitor, therapy, drug

## Abstract

Amyotrophic lateral sclerosis (ALS) is the most common neurodegenerative disorder of motor neurons in adults, with a median survival of 3–5 years after appearance of symptoms, and with no curative treatment currently available. Frontotemporal dementia (FTD) is also an adult-onset neurodegenerative disease, displaying not only clinical overlap with ALS, but also significant similarities at genetic and pathologic levels. Apart from the progressive loss of neurons and the accumulation of protein inclusions in certain cells and tissues, both disorders are characterized by chronic inflammation mediated by activated microglia and astrocytes, with an early and critical impact of neurodegeneration along the disease course. Despite the progress made in the last two decades in our knowledge around these disorders, the underlying molecular mechanisms of such non-cell autonomous neuronal loss still need to be clarified. In particular, immune signaling kinases are currently thought to have a key role in determining the neuroprotective or neurodegenerative nature of the central and peripheral immune states in health and disease. This review provides a comprehensive and updated view of the proposed mechanisms, therapeutic potential, and ongoing clinical trials of immune-related kinases that have been linked to ALS and/or FTD, by covering the more established TBK1, RIPK1/3, RACK I, and EPHA4 kinases, as well as other emerging players in ALS and FTD immune signaling.

## 1. Pathological and Molecular Features of ALS and FTD

Amyotrophic lateral sclerosis (ALS) is an adult-onset neurodegenerative disease characterized by the selective and progressive degeneration and loss of motor neurons in spinal cord, brain and brainstem, controlling voluntary muscles [1]. With an incidence of 1–2:100,000, ALS is currently incurable and ultimately fatal, and once symptoms arise it is typically lethal within the next 5 years [2,3]. There is substantial heterogeneity in clinical manifestations, and most cases are considered sporadic as they occur without a known cause or a known family history. However, even though clinically indistinguishable from sporadic ALS, about 5–10% of cases are caused by genetic mutations and referred to as familiar ALS [3]. The pathophysiological processes underlying ALS are currently believed to be multifactorial, reflecting a complex interaction between genetic and environmental factors [4]. Multiple mutations have been located within four major ALS-associated genes, namely *C9orf72*, *SOD1*, *TARDBP* (encoding the TDP-43 protein) and *FUS*, in addition to at least 40 other genes [5].

A key pathological hallmark of ALS is the accumulation of misfolded TDP-43 and, in some cases, SOD1 or FUS proteins. These aberrant protein aggregates are currently thought to cause neuronal damage due to a loss of the native protein’s function and/or by a gain of toxic function exerted through various possible mechanisms [6]. Indeed, different pathogenic processes are supported by the accumulated data from in vitro, ex vivo and in vivo studies: mitochondrial dysfunction, reactive oxygen species (ROS) associated oxidative stress, dysregulated proteostasis, impaired autophagy, activation of ER-induced cell death, impaired stress granule formation, and altered RNA metabolism and/or processing [3,6]. Apart from these general cytotoxic mechanisms, neuroinflammation—a non-cell autonomous mechanism of neurodegeneration and neuronal loss—is currently considered a key player in the onset and progression of ALS and other neurodegenerative diseases [7].

Frontotemporal degeneration (FTD) is a fatal type of dementia characterized by atrophy of the frontal and temporal brain lobes, and is associated with behavioral and personality changes [3,8]. Mutations in three genes, namely *MAPT* (encoding tau), *GRN* and *C9orf72*, agglutinate the majority of familial FTD cases. Remarkably, approximately 15% of patients display motor symptoms and up to 50% of ALS patients develop frontal lobe dysfunction that may be associated with cognitive and behavioral impairment [9]. Even though ALS and FTD are thought to be at different ends of a pathological spectrum and have traditionally been considered two independent clinical entities, many patients present both conditions (ALS-FTD) [3,8]. Moreover, it is increasingly recognized that they partially overlap in their clinical manifestations and display certain common genetic origins and pathophysiological mechanisms [8,10]. For example, a number of genes have been identified as causes or risk factors of both ALS and FTD, such as *TARDBP*, *C9orf72*, *VCP*, *UBQLN2*, *SQSTM1* and *TBK1*, among others. Interestingly, several of these genes are implicated in specific molecular pathways, namely RNA regulation, autophagy, vesicle and inclusion formation, and immunity [5,10]. Furthermore, the accumulation of ubiquitinated protein aggregates in degenerating neurons and other CNS cell types, particularly cytoplasmic TDP-43 species and C9orf72 dipeptide-repeat protein (DPR) aggregates, are also a common denominator in tissue samples from ALS and FTD patients, both familial and sporadic [10].

Another common feature of ALS and FTD, which is shared with virtually all neurodegenerative diseases, is the build-up of sustained neuroinflammation in affected regions of the CNS [3]. Peripheral immune cells, particularly T cells and other non-CNS-residing immune cells, are known to contribute to disease progression in some neurodegenerative diseases, and we have recently found that disease-like protein aggregates are capable of eliciting responses both from innate and peripheral immune cells [11,12]. However, the involvement of peripheral immunity in ALS or FTD is still poorly understood, and the bulk of evidence points to activation of innate immune signaling pathways in microglia and astrocytes resident in the CNS as a very early event in the pathological process [3,13]. In ALS, even though both neuroinflammation and systemic inflammation are consistently present in ALS patients—and, according to animal models, since the early stages of disease—whether these phenomena are an outcome of disease, or instead play a contributory or even causative role in pathogenesis, is still under debate. Importantly, activated microglia and astrocytes in animal models of ALS at the initial stages show anti-inflammatory and neuroprotective profiles, whereas in the late stages they display proinflammatory and neurodegenerative features [4], supporting the notion that neuroinflammation has a central role in the onset, exacerbation, progression, and stages of the disease [3,13].

## 2. Importance of Immune-Related Kinases in ALS and FTD

**Figure 1:**
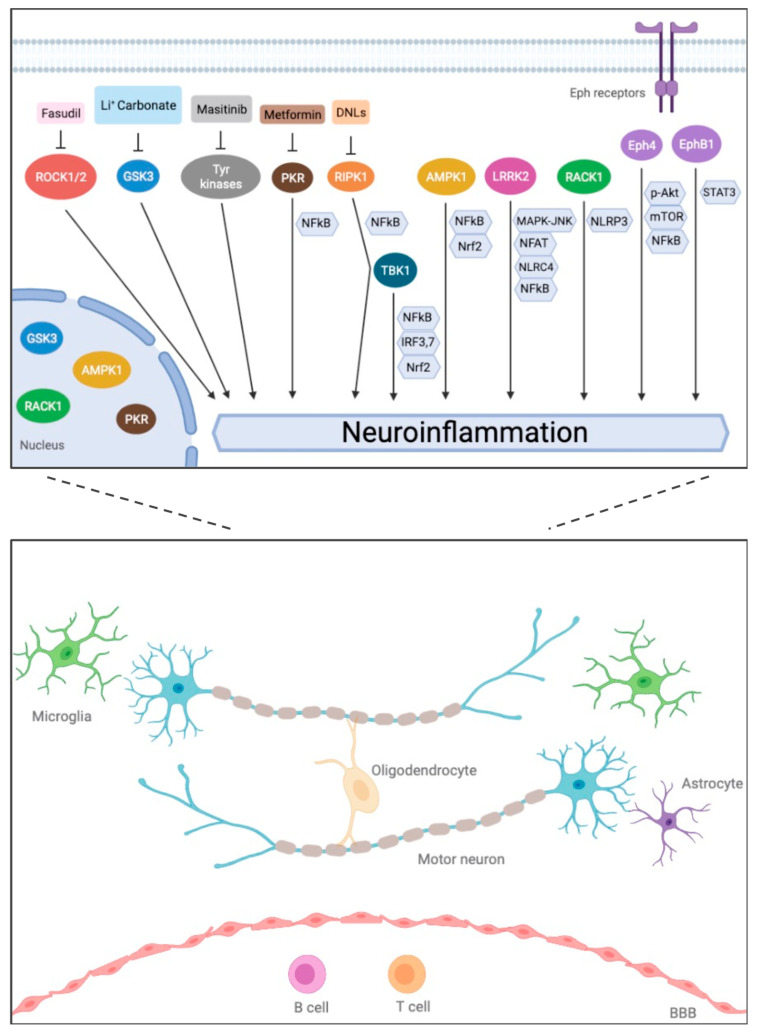
Immune-related kinases involved in ALS/FTD physiopathology. Schematic overview of immune-related kinases that have been implicated in in ALS or FTD, based on in vitro, ex vivo, in vivo or genetic studies. (**Top**): Signaling kinases known to display immune-related functions (ovals) and the identified signaling pathways in which they participate (hexagons) from various inflammation-based disease models. Specific kinase inhibitors (rectangles) that have been used to modulate some of these kinases are also shown. (**Bottom**): Different cell types located within the CNS or in the periphery, through which immune-related kinases may play a role in ALS/FTD pathophysiological processes.

Remarkably, the accumulated evidence for ALS indicates that, regardless of the molecular mechanisms of injury operating on neuronal cells, different cellular pathways that may be triggered converge, leading to a neurodegenerative inflammatory cascade [4]. At the same time, cascades of different processes could result in motor neuron death, including cell-autonomous and non-cell autonomous mechanisms, regardless of the exact cause of each ALS case [3]. Either way, neuroinflammation is currently recognized as a major contributor to neurodegeneration in ALS and other neurodegenerative disorders [4,7]. One of the mechanisms that could have a major impact in driving uncontrolled neuroinflammation and, ultimately, neuronal cell death in ALS and FTD, is the dysfunctional regulation of certain kinases involved in immune signaling (Figure 1).

Indeed, apart from the various studies revealing the dysregulated expression of inflammatory-related genes and proteins in ALS patient tissues [3], and the implication of several signaling kinases in the pathophysiology of ALS [14], the recent discovery of *TBK1* as an ALS-causing gene [15,16] suggests a direct link between neuroinflammation and signaling kinases in ALS and other related diseases.

## 3. Kinases and Immune Signaling in ALS and FTD Pathogenesis

### 3.1. TBK1 (TANK-Binding Kinase 1)

TBK1, a pleiotropic serine/threonine kinase, is involved in the regulation of selective autophagy and inflammatory responses via type-I interferon (IFN) signaling [17]. Two independent human sequencing studies were the first to link different mutations in the *TBK1* gene to ALS [15,16]. At the same time, three pioneering studies originally connected TBK1 mutations with FTD [15,18,19]. Thus far, several studies have identified a large number of TBK1 variations dispersed throughout the protein sequence to be a major cause of both familial and sporadic, ALS, FTD and ALS-FTD [17]. Some of them have been shown to cause a loss of kinase function [20], which resulted in rather evident abnormalities including dendritic swelling, abnormally shaped astrocytes, and p62-and ubiquitin-positive aggregates in the cerebellum, as observed in TBK1-KO mice [21]. However, the majority of ALS-associated TBK1 variants are missense mutations and their contribution to ALS pathogenesis still needs to be elucidated [17]. Interestingly, post-mortem brain analyses from ALS/FTD patients carrying TBK1 mutations have revealed cytoplasmic or perinuclear TDP-43-positive and p62-positive inclusions [16,19,22]. These findings may not seem surprising as TBK1 has been implicated in autophagy [17], while an ALS-associated TBK1 variant was unable to promote TDP-43 autophagic degradation dependent on p62 phosphorylation [23].

TBK1 is highly expressed in neurons among other cell types, and its expression and activation has also been observed in microglia [17,24]. TBK1 participates in immune signaling pathways by acting as an essential component of the type I IFN response (reviewed in [25]). Indeed, during the antiviral innate immune response, TBK1—which belongs to the IKK-kinase family—becomes activated and phosphorylates the downstream transcription factors IRF3 and IRF7, which dimerize, translocate to the nucleus, and initiate gene expression of IFNα and IFNβ cytokines. Recent studies in vitro have shown that ALS-associated TBK1 variants retain different levels of kinase activity and display a range of signaling efficiencies leading to IRF3 phosphorylation [26]. In addition, results from another in vitro study indicate that, whereas wild-type TBK1 promotes the NF-kB pathway and suppresses Nrf2 signaling, the ALS-associated p.G175S kinase-deficient, TBK1 mutant does not [23].

In addition, an essential role of TBK1 in inhibiting RIPK1-dependent apoptosis and inflammation, via phosphorylation of a particular Thr residue downstream of TNF receptor 1 (TNFR1), was demonstrated [27]. Remarkably, the study further showed that, in TBK1 deficient mice, the reduced myeloid expression of TAK1—an endogenous RIPK1 inhibitor—promoted the key ALS/FTD phenotype hallmarks, including microgliosis, TDP-43 aggregation, neuronal loss, and behavioral deficits [27]. Moreover, global heterozygous deletion of *TBK1* modelling the TBK1 loss-of-function mutations found in ALS/FTD in humans, was seen to exert a dual and stage-specific effect in SOD1^G93A^ mice, by initially preponing muscular denervation and clinical onset, but reducing microglial neuroinflammation and decelerating disease progression at later stages [28]. Interestingly, a recent study based both on general knockout and motor-neuron-selective deletion of TBK1 in SOD1^G93A^ mice proposed that the observed reduction in gliosis in spinal cord is actually a non-cell autonomous result of selective loss of TBK1 in motor neurons [29], whereas decreased TBK1 kinase activity in all cells reduces the phosphorylation of IRF3 in SOD1^G93A^ mice, resulting in suppression of IFN-stimulated genes in glial cells and leading to slowing of neurodegeneration [29].

### 3.2. RIPK1 (Receptor-Interacting Kinase 1)

RIPK1 is a death-domain containing Ser/Thr kinase which functions as a key regulator of cell death—including necroptosis and RIPK1-dependent apoptosis—and inflammatory responses, and has been implicated in a number of CNS diseases [30,31]. In particular, RIPK1 kinase activity has also been shown to promote inflammatory responses under caspase-deficient conditions [32]. Even though there is no causal evidence linking RIPK1 mutations to ALS, this gene was shown to mediate axonal degeneration by promoting inflammation and subsequent oligodendrocytes necroptosis in ALS [33], and a proinflammatory role of RIPK1 kinase activity in the CNS in association with TBK1 was demonstrated, as mentioned above [27]. Importantly, a recent study based on single-cell RNA sequencing has identified a RIPK1-regulated inflammatory microglial (RRIM) subset in early-stage SOD1^G93A^ mice [34]. Differently from Disease-Associated Microglia (DAM), the RRIM state was characterized by up-regulation of classic proinflammatory pathways, and was found to peak at an earlier time point in the ALS model, compared to the DAM phenotype [34].

### 3.3. Eph Receptors (Ephrin Receptor Family)

The Eph receptors of tyrosine kinases, in combination with their ephrin ligands, constitute a bidirectional signaling mechanism known to mediate neuron–glia crosstalk and to undergo expression alterations following neuronal injury and neurodegeneration [35]. A link for these receptors with ALS was originally established when Epha4 was identified as a modulator of motor neuron degeneration and disease progression in fish and mouse disease models [36]. In addition to its role in the regulation of axonal growth and remodeling, Epha4 was found to function as a mediator of inflammation in spinal cord and traumatic brain injuries [37,38], potentially via the mTOR, p-Akt and NF-kB pathways [38].

Epha4-specific nanobodies [39], an EphA4 receptor soluble antagonist [40] and selective Epha4-binding compounds [41], were developed to treat ALS and delivered encouraging results in delaying disease progression in the SOD1^G93A^ model. However, pre-symptomatic lowering of Epha4 levels in ALS mice, ubiquitously or specifically in CNS, by genetic abrogation or by administration of antisense oligonucleotides (ASOs) did not show a significant improvement in motor function, disease onset, or survival [42,43]. This probably reflects a highly complex mechanism of Epha4, or the ephrin system in general, in ALS pathogenic processes [14]. On the other hand, a study based on the SOD1^G93A^ mouse model and on human iPSC-derived astrocytes provided evidence that EphB1-mediated signaling can induce astrocytic STAT3 activation, invoking a protective transcriptional profile [44]. Importantly, the authors also demonstrated that such EphB1-induced pathways are disrupted in ALS, resulting in impaired astrocyte activation and failure in response to neuronal injury [44].

### 3.4. RACK1 (Receptor of Activated Protein C Kinase 1)

RACK1, an intracellular protein receptor for protein kinase C (PKC), has been associated with ALS as it was found to co-localize with cytoplasmic TDP-43 in spinal motor neurons in patients [45]. Additionally, the same study revealed that RACK1—which functions as a ribosomal scaffold for several proteins—mediates TDP-43 binding to ribosomes and polysomes and recruitment of TDP-43 to stress granules [45]. Interestingly, RACK1 was also shown to modulate microglial resistance against LPS-induced inflammatory injury [46]. Moreover, non-ribosomal RACK1 was recently shown to mediate the activation of the inflammasome system—intracellular multiprotein complexes belonging to innate immunity that enable a rapid and powerful inflammatory response against multiple stimuli—by serving as a key NLRP3 interacting protein that promotes the NLRP3 active conformation and inflammasome assembly [47].

### 3.5. AMPK (AMP-Activated Protein Kinase) and LRRK2 (Leucine-Rich-Repeat Kinase 2), New Immune Signaling Kinases in ALS/FTD

Certain kinases with known functions in immune signaling have been recently involved in ALS or FTD based on studies using preclinical models and samples from patients. AMPK, a Ser/Thr kinase which is ubiquitously expressed in mammalian cells, including neurons and immune cells in the brain, is one such case. Intriguingly, studies have reported enhanced AMPK activation in spinal cord from SOD1^G93A^ mice and spinal motor neurons from ALS patients, but diminished AMPK activation in spinal cord and brain from TDP-43 mutant mice (reviewed in [48]). AMPK functions as a master energy sensor for regulating cellular metabolism and maintaining energy homeostasis, and metabolic and energetic dysfunction are well-documented features in ALS [48]. In addition, AMPK plays a prominent role—shared with the mTOR complex 1 (mTORC1) kinase—in initiation of autophagy by activating the ULK1 complex. Indeed, there is mounting evidence that the autophagy-lysosome pathway is compromised, via multiple proposed mechanisms, of C9orf72 and TDP-43 ALS-FTD models (reviewed in [49,50]). Importantly, accumulating data based on cell culture studies and rodent animal models, also implicate AMPK in the regulation of neuroinflammation by promoting the shift of microglial activation from pro-inflammatory to anti-inflammatory states, via inhibition of NF-kB signaling and enhancement of the Nrf2 pathway (reviewed in [51]).

A recent genome-wide association study assessing the genetic overlap between FTD-related disorders and inflammatory diseases identified LRRK2 and TBK-binding protein 1 (TBKBP1) as FTD association candidates [52]. Two other genetic studies had previously proposed an association between LRRK2 mutations—well known to be a cause of Parkinson’s and Crohn’s diseases—and FTD [53,54] and a recent NGS-bioinformatics-based analysis of a sporadic FTD cohort, further supported this association [55]. LRRK2 is a multi-domain protein bearing GTPase and Ser/Thr kinase catalytic activities, as well as diverse protein interacting regions. LRRK2 is highly expressed in immune cells and has been widely implicated in diverse immune-related pathways in peripheral and central immune cells, including microglia (reviewed in [56,57]). In particular, results from studies based on inflammatory and neurodegenerative disease models indicate that LRRK2 promotes neuroinflammation by a number of mechanisms that may be dysregulated in microglia and other immune cells, including the activation of MAPK-JNK signaling [58,59,60], suppression of the NFAT cytokine-regulating transcription factor [61,62], dectin-1-mediated C-type lectin signaling [63], phosphorylation and activation of NLRC4-inflammasome [64], and promoting the NF-κB pathway by controlling the NF-κB p50 inhibitory signal and by negatively regulating the activation of protein kinase A (PKA), an inhibitor of NF-κB signaling [65,66].

Apart from its prominent role in immune signaling, accumulating evidence also supports a role of LRRK2 in autophagy and the lysosome pathway in immune cells, particularly in the context of inflammatory responses in monocytes, astrocytes and macrophages (reviewed in [57]). Interestingly, LRRK2 was found to interact with RIPK1 and to enable the formation of a distinct and essential ubiquitinated RIPK1 intermediate along the RIPK1-dependent apoptosis pathway [67,68]. However, possibly owing to the very recent connection established between LRRK2 and ALS/FTD, no study has thus far interrogated the possible role of LRRK2 in ALS/FTD immune dysregulation.

## 4. Other Emerging Signaling Kinases in ALS/FTD

Protein kinase RNA-activated (PKR) is a central intracellular stress sensor that can be activated by a variety of stimuli, particularly dsRNA, leading to autophosphorylation and resulting in proinflammatory responses via NF-kB, p38 and c-Jun N-terminal kinase (JNK) signaling, as part of the classical innate immunity to viral infection [69]. Repetitive element (RE) transcripts, which are prone to form dsRNA species, were found at higher levels in ALS patients’ brains [70], while mechanistic relationships between dysregulated TDP-43 function or C9orf72-repeat expansion, and increased transposable RE transcripts, have been suggested [70,71]. Interestingly, a recent study based on astrocyte primary cell cultures demonstrated that knocked-down TDP-43 leads to higher RE transcript and dsRNA levels [72]. Moreover, results from double knockdown assays support a mechanism by which PKR—which had been detected at higher levels in spinal cord samples from ALS patients [73]—mediates astrocyte activation and the ‘reactive’ proinflammatory phenotype resulting from TDP-43 downregulation [72]. On the other hand, a recent study demonstrated that PKR highly regulates repeat-associated non-AUG (RAN) translation [74], an aberrant mechanism reported in various ‘repeat expansion diseases’ such as C9orf72 ALS and FTD [75,76,77]. Furthermore, the study showed that repeat expansion RNAs, including ALS/FTD ‘C9 repeats, increase active/phosphorylated PKR (p-PKR) levels in vitro and in vivo in mice, while higher p-PKR levels were detected in hippocampal tissue from C9orf72 ALS patients [74].

Recently, as a result of our study of the inflammatory response in microglial cells exposed to TDP-43 aggregates, we identified MAPK/MAK/MRK overlapping kinase (MOK) as a protein that strongly interacts with cytoplasmic TDP-43 aggregates [78]. Moreover, MOK was found to alter its phosphorylation state in critical residue Tyr161, both in primary microglia and organotypic spinal cord cultures [78]. MOK is an atypical Ser/Thr signaling kinase that belongs to the MAPK superfamily [79], and which has been predicted to play an important role in diverse physiological and pathological processes [80].

## 5. Immune Kinase Modulation for ALS and FTD in Disease Managing and in Clinical Trials

Thus far, only two drugs have been approved for therapeutic treatment of ALS: Riluzole, by both the FDA and EMA agencies and also approved for FTD [81], and Edaravone, authorized more recently by the FDA [82]. Riluzole—via poorly understood mechanisms—displays activity against glutamatergic excitotoxic and non-excitotoxic oxidative neuronal injury, and has been used extensively in the clinic for 25 years, prolonging median survival by only two to three months in ALS patients [83]. Interestingly, Riluzole was later found to be a direct inhibitor of protein kinase C (PKC) [84], a kinase family well-known to play a key role in the development and effector functions of major immune cell types, and implicated in both innate and adaptive responses [85]. Because of the limited therapeutic power of these drugs, there is a clear need to identify novel molecular targets that could be potentially modulated in a specific and effective manner for the treatment of ALS and FTD. Currently, a few immune kinase inhibitors are being tested in clinical trials (Table 1).

### 5.1. Masitinib: A Receptor Tyr Kinase Inhibitor

Masitinib is a selective tyrosine kinase inhibitor [86] with neuroprotective and anti-inflammatory capability demonstrated in a number of preclinical studies [86,87,88,89]. Masitinib was shown to target a limited number of kinases known to be involved in cancer and inflammatory diseases, at safe doses [86] and was later demonstrated to reduce neuroinflammation by controlling microgliosis and counteracting mast cell degranulation [87,90]. The compound is thought to exert neuroprotection via its immunomodulatory properties through targeting microglia, macrophages, mast cells and neutrophils, and implicating both central and peripheral nervous systems [91,92]. Importantly, oral administration of this compound showed a 25% delay in disease progression in a recent phase II/III clinical as an add-on therapy to Riluzole in ALS patients [92]. Masitinib has now entered a phase III clinical trial, which is currently ongoing.

### 5.2. Fasudil: A ROCK1/2 (Rho-Kinase 1/2) Inhibitor

ROCK is a Ser/Thr kinase that is expressed as two homologues, ROCK1 and ROCK2, and whose activity is modulated by Ras-superfamily upstream regulators, namely the Rho GTPases RhoA and RhoC. ROCK phosphorylates a number of targets involved in regulation of a variety of crucial cellular processes including cell motility, apoptosis and cell survival in different cell types, including neurons and microglia (reviewed in [93,94]). In the latter, the ROCK pathway contributes to defining microglial phenotype by regulating microglial migration, phagocytosis and the release of inflammatory cytokines [95,96]. While abnormally high levels of ROCK were detected in skeletal muscle biopsies from sporadic ALS patients [97], pre-symptomatic administration of Fasudil to SOD1^G93A^ mice improved survival and motor performance [98].

At a pathological level, motor neuron degeneration was shown to be partially suppressed and neuromuscular junction remodeling, enhanced. Interestingly, while the number of astroglial cells was reduced, Fasudil treatment led to increased microglia numbers and shifted activation profile from the neurotoxic M1 to the protective M2 phenotype [98], in line with previous in vitro and in vivo studies in other pathophysiological contexts [99,100]. Furthermore, oral administration of Fasudil to symptomatic SOD1^G93A^ mice resulted in improved motor behavior in males [101]. Still, there is currently no sufficient evidence for a direct immune-based mechanism of ROCK in ALS pathology. Instead, recent pre-clinical studies with Fasudil in SOD1 mice provide evidence for participation of ROCK dysregulation in ALS pathogenesis caused by imbalance of pro-survival Rac/pro-apoptotic Rho-ROCK signaling in motor neurons [102] and by impaired ROCK-signaling controlling axonal regeneration [103]. This compound, applied intravenously, is currently undergoing a phase II clinical trial.

### 5.3. DNL Compounds: RIPK1 Inhibitors

Based on the functions recently described for RIPK1 kinase in regulating inflammation and necroptotic cell death, specific small-molecule inhibitors have been developed as treatment candidates for chronic inflammatory diseases [104,105]. In 2017 and shortly after its initiation, a phase Ia clinical study of DNL104—a brain-penetrant RIPK1 inhibitor—was discontinued due to liver toxicity effects. A selective and brain-permeable RIPK1 inhibitor DNL747 was designed subsequently and announced in 2020 for a phase Ib/II clinical trial in ALS [105], but the study was paused when it was concluded that higher doses—which were likely to cause adverse effects according to studies with cynomolgus monkeys [106]—would be needed to improve efficacy. Importantly, the alternative compound DNL788, which appears to display a superior profile and is expected to avoid the side effects seen at high doses for DNL747, is due to enter phase II clinical testing in 2022 [107].

### 5.4. Lithium Carbonate: A GSK3α/β (Glycogen Synthase Kinase 3) Inhibitor

GSK3 has been found to be a point of convergence of many signaling pathways and to regulate, in a highly complex manner, many cellular functions through its capacity to phosphorylate a wide variety of substrates [108]. Remarkably, studies in the last two decades have revealed that GSK3 participates in several signaling pathways and is an important regulator of innate immune responses by enabling cytokine production upon TLR stimulation in both the periphery and the CNS, and of adaptive immunity by modulating the specificity and clonal expansion of T and B cells [108,109]. Based on its role as a major tau kinase, inhibition of GSK3 with lithium—the gold-standard treatment for bipolar disorder—has been tested for the treatment of tauopathies, including AD and subtypes of FTD. In cultured cells and in transgenic mice, lithium-induced GSK3β inhibition was seen to reduce tau phosphorylation, increase the binding of tau to microtubules and promote microtubule assembly [110,111]. Interestingly, Li+ has been shown to rescue autophagy failure observed in ALS/FTD and, in recent years, lithium administration has been assessed in clinical trials in ALS, thus far with debatable results [112]. Of note, a phase II clinical trial with low-dose lithium (against placebo) for the treatment of behavioral symptoms in FTD is currently ongoing [113].

## 6. Immune Kinase Modulators in the Pipeline towards ALS/FTD Therapy

### 6.1. Metformin: A PKR Inhibitor

Based on results obtained in vitro which showed that metformin—a widely prescribed drug for type 2 diabetes treatment, known to activate the AMPK pathway [114]—inhibits PKR and reduces the levels of several RAN proteins in mammalian cells, the effect of this drug was tested in vivo by using C9-BAC mice, a C9orf72-based pre-clinical model of ALS [74]. Administration of metformin at the pre-symptomatic stage resulted in lowered DPR aggregates and p-PKR levels. Moreover, as opposed to a previous study using the SOD1^G93A^ ALS model [115], treatment of C9-BAC mice with the drug up until the advanced stage led to substantially reduced astrogliosis in the motor cortex, decreased motor neuron degeneration in the spinal cord, and improved locomotive and behavioral parameters, compared to non-treated animals [74].

### 6.2. C4: An Epha4 Ligand

Following encouraging results by a study showing significant delay in disease progression in the SOD1^G93A^ mouse model [41], the brain-penetrant, high-selectivity and high-affinity small molecule EPHa4 ligand 123C4—presumably a receptor agonist—is currently being evaluated in preclinical models for ALS by Iron Horse Therapeutics (San Diego, CA, USA) [88].

## 7. Concluding Remarks

In the last few years, it has become increasingly evident that ALS and FTD share clinical, genetic, and mechanistic cues, a feature which may provide hints on their elusive pathogenic mechanisms. A number of signaling pathways and their executing kinases—many of which are known to mediate immune functions, particularly, inflammatory responses—have been recently linked to ALS or FTD. Some of them have been associated genetically to these disorders, such as TBK1; others, including e.g., RIPK1, do not seem to have a direct genetic link with these disorders, but are known to participate in their pathogenic mechanisms.

An increasing number of studies in recent years point to a mechanistic link between mitochondrial and autophagy disruption in neurodegenerative disorders, including ALS and FTD. Interestingly, neuroinflammation, which has emerged as a principal determinant of neurodegeneration in these disorders, has been recently linked to both dysregulated processes [17,116], adding a possible new layer of complexity to the neuroinflammation–neurodegeneration interaction network.

Based on the growing body of knowledge generated in the last two decades in ALS and especially in AD and other related neurodegenerative diseases, a number of immune kinases have emerged as highly promising molecular targets for therapeutic intervention. Indeed, a short list of immune kinase inhibitors are currently being tested in clinical trials or are undergoing pre-clinical studies. Surely, due to a virtual lack of an effective therapy for ALS and FTD patients, further investigation towards the identification of novel therapeutic targets is warranted.

From the reviewed data, it seems obvious that, despite the progress made over the last two decades, substantial effort must still be made in the field to improve our understanding of the intricate mechanisms underlying ALS and FTD pathogenesis, including immune signaling pathways, which would prompt the identification of novel kinase therapeutic targets. In addition to a better understanding of these highly complex pathogenic processes, better stratification of patients would improve the design and outcome of clinical trials and, thereby, boost therapeutic development.

## Figures and Tables

**Table 1:** Clinical trials based on immune kinase inhibitors currently underway for ALS or FTD treatment.

Drug	Kinase Target	Study Phase	Year Launched ^1^	Code Number	Disease
Masitinib	Tyrosine kinase receptors	III	2020–2022	NCT03127267	ALS
Fasudil	ROCK	II	2020	NCT03792490	ALS
DNL747	RIPK1	Ib (paused)	2020	NCT03757351	ALS
DNL788/ SAR443820	RIPK1	II (announced)	Q1 2022	N/A	ALS
Lithium carbonate	GSK3	II (recruiting)	2021	NCT02862210	FTD

^1^ Phase starting year (according to latest update).

## Data Availability

Not applicable.
